# First report of *Borrelia burgdorferi* sensu lato in two threatened carnivores: the Marbled polecat, *Vormela peregusna* and the European mink, *Mustela lutreola* (Mammalia: Mustelidae)

**DOI:** 10.1186/1746-6148-8-137

**Published:** 2012-08-18

**Authors:** Călin M Gherman, Attila D Sándor, Zsuzsa Kalmár, Mihai Marinov, Andrei D Mihalca

**Affiliations:** 1 Department of Parasitology and Parasitic Diseases, University of Agricultural Sciences and Veterinary Medicine, Faculty of Veterinary Medicine, Calea Mănăștur 3-5, Cluj-Napoca, 400372, Romania; 2 Department of Taxonomy and Ecology, Babeş-Bolyai University, Faculty of Biology, Strada Clinicilor 5-7, Cluj-Napoca, 400006, Romania; 3 Danube Delta National Institute for Research and Development, Strada Babadag 165, Tulcea, 820112, Romania

**Keywords:** *Borrelia burgdorferi* s.s, First report, *Mustela lutreola*, *Vormela peregusna*, Romania

## Abstract

**Background:**

Lyme disease is a widespread cosmopolitan zoonosis caused by species belonging to the genus *Borrelia*. It is transmitted from animal reservoir hosts to humans through hard - ticks of genus *Ixodes* which are vectors of the disease.

**Case presentation:**

*Borrelia burgdorferi* sensu lato infection was identified in a marbled polecat, *Vormela peregusna*, and two European minks, *Mustela lutreola*, from Romania, by PCR. RFLP revealed the presence of a single genospecies, *Borrelia burgdorferi* sensu stricto.

**Conclusions:**

This is the first report of the Lyme disease spirochetes in the two mentioned hosts.

## Background

*Borrelia burgdorferi* sensu lato is the causative agent of Lyme borreliosis, the most widespread vector-borne disease in the cool-temperate regions of the Northern hemisphere. The medical importance of this pathogen is generally restricted to humans and few domestic species. However, as for many vector-borne diseases, the key to the understanding of the epidemiology of Lyme borreliosis consists in revealing the ecological relationships that exist between pathogens, vectors and wildlife hosts [1]. All 18 currently recognized genotypes [2] circulate in nature between vectors (several ticks of genus *Ixodes*) and reservoir hosts (various vertebrates) which are able to maintain and transmit the spirochetes [3].

## Case presentation

Between 2009 and 2011, 5 specimens belonging to two species of threatened mustelids were brought to the Laboratory of Parasitology and Parasitic Diseases in deep frozen state (Table 1). The dead animals were collected either as road kills (*Vormela peregusna*) or as accidental casualties of live-trapping during field studies (*Mustela lutreola*). The trapping was performed as part of biodiversity and ecology studies in the central part of the Danube Delta using box traps [4]. The accidental death of animals in live traps was caused by extreme morning frost or poor overall health status caused by floods (Kiss JB personal communication). Examination of the fur and skin did not reveal the presence of external parasites.

**Table 1:** The collection localities of threatened Mustelidae specimens used in this study

**Sample**	**Species**	Locality (County^*^)	**Coordinates**	**Date**
ML1	European mink (*Mustela lutreola*)	Canal Litcov (TL)	45°08'N 29°19'E	05.03.2010
ML2	European mink (*Mustela lutreola*)	Canal Litcov (TL)	45°08'N 29°18'E	06.03.2010
ML3	European mink (*Mustela lutreola*)	Canal Litcov (TL)	45°08'N 29°18'E	06.03.2010
VP1	Marbled polecat (*Vormela peregusna*)	Sinoe (CT)	44°37'N 28°43'E	30.11.2009
VP2	Marbled polecat (*Vormela peregusna*)	Agighiol (TL)	45°02'N 28°53'E	10.06.2011

^*^ CT: Constanţa; TL: Tulcea.

During the necropsy of these animals, tissue samples (myocardium) were collected and processed for DNA extraction (Qiagen, DNeasy Blood & Tissue Kit). An extraction blank was included in each extraction procedures to control the cross-contamination between extracts. The crude DNA was analyzed by a PCR protocol according to Priem et al. (1997) [5] using primers (Generi Biotech) for *Borrelia burgdoferi* sensu lato (5’-GGGAATAGGTCTAATTTAGCC-3’, 5’-CACTAATTGTTAAAGTGGAAGT-3’) targeting the OspA gene [6]. Each time the PCR was performed including negative control samples. The positive PCR products were further analyzed by RFLP using two restriction enzymes *Alw*l (BsPI) and *Mse*I (Tru1 I) (Fermentas), according to the manufacturers protocol. PCR analysis revealed positivity to *Borrelia burgdorferi* sensu lato in three samples (ML2, ML3 and VP1) belonging to the two host species: *M. lutreola* and *V. peregusna*.

**Figure 1:**
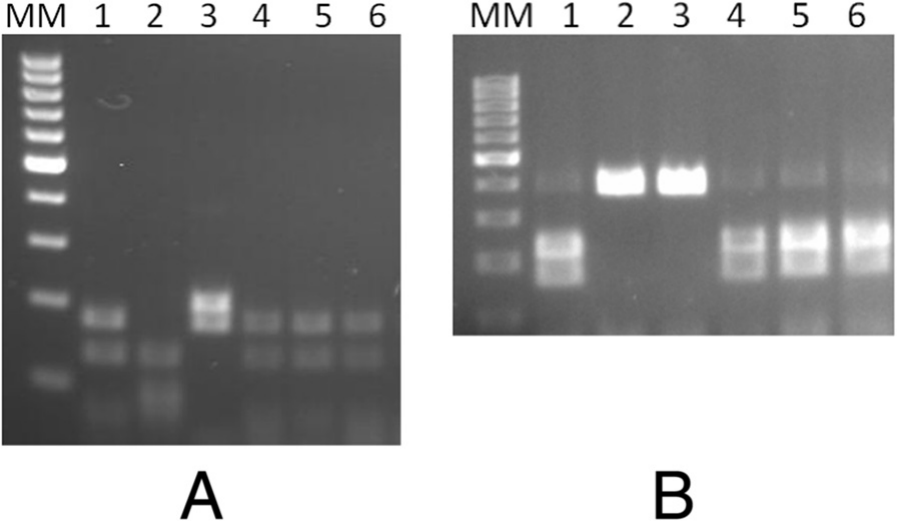
**RFLP pattern of the amplified *ospA *gene cut with MseI (Trull) (A) and Alwl (BsPI) (B): M - 100 bp Molecular ladder (Fermentas).** 1-3 – positive controls: *B. burgdorferi s.s., B. garinii, B. afzelii*, 4 - *B. burgdorferi s.s.* (*Vormela peregusna*), 5 - *B. burgdorferi s.s.* (*Mustela lutreola*), 6 - *B. burgdorferi s.s.* (*Mustela lutreola*).

Analysis of the RFLP pattern of the amplified OspA gene cut, showed bands at 183/134/74 base pairs (bp) for *Mse*I (Tru1 I) (Figure 1A) restriction enzyme and a second pattern at 227/164 bp for *Alw*l (BsPI), which is indicative of *Borrelia burgdorferi* sensu stricto (s.s.) in all three positive samples (Figure 1B).

## Discussion

*Borrelia burgdorferi* is maintained in natural cycles of infection by vector ticks and reservoir hosts. In Western and Central Europe, *Ixodes ricinus* is particularly important in the transmission of the Lyme spirochete. Moreover, *I. hexagonus* has been experimentally confirmed as a vector [7], but was also shown to be important in secondary cycles of *B. burgdorferi* transmission [1,8]. Both of them, together with other congeneric ticks, are well represented in mustelids (Table 2). Although ticks are commonly found on mustelids, *B. burgdorferi* s.l. infection is rarely reported. The scarcity of reports can be explained either by the fact that the infection is rare or, most probably by the lack of studies on this topic.

**Table 2:** Check-list of Ixodid tick species associated with mustelids

**Ticks species**	**Mustelid hosts**
*I. acuminatus*	*Mustela nivalis*[22], *Mustela putorius*[22], *Mustela vison*[23]
*I. angustus*	*Mustela erminea*[24]
*I. cookei*	*Mustela frenata*[25], *Mustela nivalis*[26], *Mustela vison*[26,27]
*I. crenulatus*	*Martes foina*[28], *Meles meles*[24], *Mustela erminea*[29],
	***Mustela lutreola***[30], *Mustela nivalis*[28], *Mustela putorius*[31], *Mustela vison*[23],
*I. gregsoni*	*Martes americana*[32], *Martes pennanti*[33], *Mustela* sp. [32], *Mustela vison*[32,33]
*I. hexagonus*	*Martes foina*[13,31,34-36], *Mustela putorius*[31,35,36],
	*Mustela erminea*[29,31,35-37], *Mustela nivalis*[31,38]*,*
	*M. putorius furo*[31,39], *Meles meles*[34-36], *Mustela vison*[23]
*I. muris*	*Mustela vison*[40]
*I. nipponensis*	*Mustela sibirica*[41]
*I. pacificus*	*Mustela frenata*[42], *Taxidea taxus*[42]
*I. persulcatus*	*Martes flavigula*[37], *Martes martes*[37], *Meles meles*[37], *Mustela erminea*[37], *Mustela nivalis*[37], *Mustela putorius*[37], *Mustela sibirica*[37]
*I. ricinus*	*Martes foina*[13,22,35,37], *Martes martes*[37], *Meles meles*[14,35,37], *Mustela erminea*[14,29], ***Mustela lutreola***[37],
	*Mustela nivalis*[37], *Mustela putorius*[14,37], *Mustela vison*[23]
*I. rugicollis*	*Martes* sp. [43]
*I. scapularis*	*Mustela erminea*[16], *Mustela frenata*[25]
*I. tanuki*	*Martes flavigula*[44]
*I. texanus*	*Mustela frenata*[25]
*I. ventalloi*	*Mustela nivalis*[38]

Several authors have previously reported the presence of *Borrelia burgdorferi* s.l. in *Ixodes* ticks removed from mustelids: European badger (*Meles meles*) [9], American mink (*Mustela vison*) [10], weasel (*Mustela nivalis*) [11], beach marten (*Martes foina*), European polecat (*Mustela putorius*), and stoat (*Mustela ermina*) [12]. In Italy, none of the *I. hexagonus* and *I. ricinus* collected from beech marten was positive by PCR for *B. burgdorferi* s.l. [13].

An interesting study from Switzerland, where questing ticks were analyzed for the host of the previous feeding stage by Reverse Line Blotting revealed the presence of *Borrelia burgdorferi* s.l. DNA in specimens positive for *Meles meles* and *Mustela putorius* probes. The authors suggest the role of mustelids as reservoir hosts for *Borrelia burgdorferi*[14].

Other authors examined mustelids for the presence of anti-*Borrelia burgdorferi* s.l. antibodies. One least weasel caught in forests of the western part of France was seropositive for *B. burgdorferi* at 1/50 antibody titer [15], however stoats from Canada infested with *I. Scapularis* (the most important vector of Lyme disease in North America) were seronegative [16].

To our knowledge, there is a single study showing the presence of *B. burgdorferi* s.l. DNA in tissues of mustelids. McDonald and Lariviere (2001) [17] found the infection in 10 of 45 stoats from Great Britain. However, no information was provided on the tissues examined or the genospecies identified. Here we report the first identification of *B. burgdorferi* s.l. from the myocardium of a marbled polecat and two European minks. The Marbled polecat, *Vormela peregusna* is widespread from Southeastern Europe to Russia, China and northern Africa [18]. It is listed by IUCN as vulnerable. The European mink, *Mustela lutreola* is a semi-aquatic species of mustelid native to Europe and it is listed by IUCN as Critically Endangered. Its current distribution includes small isolated populations in northern Spain, western France and Eastern Europe (Latvia, Estonia, Belarus, Ukraine, central regions of European Russia, the Danube Delta in Romania and northwestern Bulgaria) [19]. In Romania, both species are present in the Southeastern part of the country, Dobrogea [20,21].

## Conclusions

Detection of Lyme spirochete is relatively rare in mustelids, suggesting the limited role of these hosts in natural foci. The detection of pathogens in tissues or in ticks feeding on various vertebrates does not confer to these hosts the status of reservoir host, but rather carrier hosts. Hence, although it has been suggested that mustelids are reservoir hosts for *B. burgdorferi* s.l., experimental transmission is required to clarify this aspect.

## Competing interests

All authors have seen and approved the manuscript and declare that they have no competing interest.

## Authors’ contributions

CMG conceived the study and drafted the manuscript. ADS made major contribution to the study design, identified the mustelid species and contributed to the writing of the manuscript. JK performed PCR and RFLP. MM had trapped the mustelids. ADM contributed to the writing of the manuscript. All authors read and approved the final manuscript.

## Financial support

This study was funded from grant IDEI-PCCE CNCSIS 84, 7/2010, while ADS was supported by a research grant POSDRU/88/1.5/S/60185.
